# Interpersonal Synchronization of Autonomic Physiology via Mere Visual Contact

**DOI:** 10.1111/nyas.70322

**Published:** 2026-06-16

**Authors:** Atesh Koul, Giacomo Novembre

**Affiliations:** ^1^ Neuroscience of Perception and Action Lab Italian Institute of Technology Rome Italy

**Keywords:** autonomic physiological signals, social cues, social interaction, spontaneous behavior, synchrony

## Abstract

Interpersonal physiological synchrony (IPS)—the temporal alignment of autonomic physiological signals—is emerging as a prominent feature of social interaction. IPS can predict complex social phenomena such as relationship quality or social bonding. Yet, the origins of IPS remain poorly understood. Here, we investigated whether and how IPS can emerge spontaneously through mere visual contact. Thirty‐eight dyads of familiar participants were asked to face each other without speaking or making coverbal gestures. IPS was measured using four autonomic signals—heart rate, skin conductance, respiration rate, and pupil diameter—and quantified as the Pearson correlation between dyad members’ signals. We found that visual contact was sufficient to synchronize all physiological signals in real dyads, compared with chance‐level synchrony in surrogate dyads. Notably, heart rate and pupil diameter IPS were particularly vision‐dependent, showing significant reductions when visual contact was prevented. Furthermore, examining the link between IPS and spontaneous social cues revealed that heart rate IPS co‐occurred with synchronized body movements, whereas pupil diameter IPS co‐occurred with synchronized smiling. These findings show that IPS can emerge spontaneously through mere visual contact and is linked to the exchange of social cues. They position IPS as a robust, self‐organizing phenomenon that occurs naturally in social interactions.

## Graphical abstract

Dyads merely looking at each other synchronize autonomic physiology, including heart rate, pupil diameter, breathing rate, and skin conductance. Synchrony in some of these measures co‐occurs with synchrony of spontaneous behaviors such as body movement and smiling. These findings position interpersonal physiological synchrony as a robust phenomenon that can emerge spontaneously through visual cues.

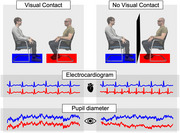

## 1 Introduction

Interpersonal physiological synchrony (IPS)—the temporal alignment of autonomic physiological signals—is emerging as a prominent feature of social interaction [1, 2, 3]. When two or more individuals interact, several distinct physiological signals have been shown to synchronize interpersonally. These include heart rate [4, 5, 6], skin conductance [7, 8, 9, 10], respiration rate [11, 12, 13], body temperature [14, 15], and pupil size [16, 17]. For instance, heart rate and skin conductance have been shown to synchronize when romantic partners discuss negative life events [18]. Similarly, respiration rates between a therapist and a client synchronize during a therapy session [8].

Physiological synchrony has been reported to emerge across a wide range of social contexts [1]. In some studies, it was reported to predict complex social phenomena such as relationship quality, social bonding, coregulation, or empathic alignment [1, 19, 20, 21, 22, 23]. Yet, to date, much of the existing research on IPS has focused on contexts where individuals are explicitly instructed to perform a social task. These tasks range from playing a game [24, 25] to solving a problem [26, 27], discussing personal topics [28, 29, 30], drumming [31, 32], or singing [12, 33]. This focus on structured contexts and instructed tasks leaves the origins of IPS unclear. In fact, these tasks often conflate several factors that may contribute to the emergence of synchronization, ranging from basic visual, auditory, or haptic contact, up to higher‐level cognitive processes related to the implementation of joint actions, verbal communication, or body gestures. Which of these is sufficient for IPS to emerge?

Studying human behavior in spontaneous and unstructured social settings might shed light on the origins of IPS, for example, by identifying what are the minimal conditions that are sufficient for this phenomenon to occur. Yet, very little research has investigated this. This is surprising considering that (1) IPS can be observed even in early infant−caregiver free interactions [34, 35], that is, when infants are not yet capable of performing an instructed social task, and (2) adults’ neural [36] and behavioral measures [37] have been shown to synchronize interpersonally through mere visual contact.

**FIGURE 1:**
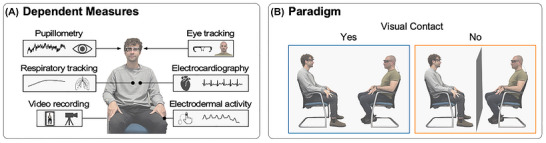
Dependent measures and experimental paradigm. (A) Dependent measures. We simultaneously measured participants’ autonomic physiological signals: heart rate, skin conductance, respiration, and pupil diameter. We also used video recordings and eye tracking to estimate the time course of spontaneous social behaviors: body movement, eye gaze, and smiling. (B) Experimental paradigm. Thirty‐eight dyads of healthy participants were seated facing each other, while their physiological signals and social behaviors were recorded over the course of 2‐min trials. Participants were only asked to look toward their partner, to behave spontaneously, and were not permitted to speak or to make coverbal gestures. Visual contact between partners was experimentally manipulated under two conditions: Vision and No Vision. In the No Vision condition, a screen was placed between the participants to prevent visual contact.

Here, we investigated whether and how physiological synchrony might emerge through mere visual contact. We combined data from two previous studies for which 38 dyads behaved spontaneously while facing each other (without speaking or making coverbal gestures; Figure 1A). We simultaneously measured four distinct autonomic physiological signals: heart rate, skin conductance, breathing rate, and pupil diameter (Figure 1A). Furthermore, we combined video recordings and deep‐learning‐based techniques to estimate the time course of three spontaneous social cues: body movement, eye gaze, and smiling.

We hypothesized that mere visual contact would be sufficient for IPS to emerge. If so, we also hypothesized that the reciprocation of social cues would co‐occur with IPS. To test the first hypothesis, we compared IPS across conditions during which the dyads could or could not see each other (Figure 1B), as well as across real and surrogate dyads (i.e., pairing the datasets of individuals who did not interact with each other). For the second hypothesis, we generated computational models that tested the association between synchronized physiological signals and synchronized social cues.

## 2 Materials and Methods

### 2.1 Participants

In the current study, we combined data from two previously collected datasets, resulting in a total of 38 dyads (i.e., 76 participants). The sample size was not determined a priori—each dataset's sample size was guided by prior work in their corresponding fields [38, 39, 40]. Importantly, the final combined sample is consistent with the sample sizes commonly reported in prior work on physiological synchrony in adults [41, 42]. The first dataset (published in [36]) included 23 dyads (mean age 21.43 years; range 18–30 years), while the second dataset (unpublished data) included 15 dyads (mean age 23.33 years; range 20–30 years). In both datasets, participants forming a dyad were familiar with each other being friends (n = 29 dyads), romantic partners (n = 8 dyads), or siblings (n = 1 dyad). While previous studies have reported IPS in both familiar [4, 24, 43, 44] and unfamiliar [45, 46, 47] pairs of participants, IPS among familiar individuals has been suggested to be more robust [48].

All participants had normal or corrected‐to‐normal vision and no history of psychological or neurological disorders. They provided written informed consent and were each compensated with 25 Euros for their participation. All experimental procedures were approved by the local ethical committee and were carried out in accordance with the principles of the revised Helsinki Declaration (World Medical Association General Assembly, 2008).

### 2.2 Original Experimental Designs

The two datasets originate from a multiperson electroencephalography (EEG) study and a multiperson transcranial alternating current stimulation (tACS) study. The first study entailed a 2 × 2 factorial design, with manipulations of visual contact (available or obstructed using an occluder separating the two participants—see Figure 1B) and spatial distance (being either 1 or 3 m). The second study entailed a 2 × 3 factorial design, with manipulations of visual contact (available or obstructed using an occluder separating the two participants) and relative phase of the brain stimulation delivered across the two participants forming a dyad (in‐phase, anti‐phase, and sham). Because we did not observe effects of brain stimulation on dyadic behavior, the three conditions were merged here. Likewise, the conditions entailing manipulations of spatial distance from the first study were merged. This led to two datasets including the same manipulation of visual contact.

Within each trial, participants forming a dyad were asked to sit face‐to‐face and behave spontaneously, without talking or producing coverbal gestures. Notably, this paradigm does not entail providing participants with any shared stimulus or task (cf. [49, 50, 51]), thereby minimizing the likelihood of synchrony arising from common external input, that is, stimulus‐driven synchrony [52, 53]. At the same time, visual contact inherently involves reciprocal sensory exchange, meaning that synchrony may still arise through perceptual entrainment or sensorimotor coupling between partners. The duration of each trial was 120 s (i.e., 2 min). All participants underwent three repetitions (i.e., trials) of each experimental condition, resulting in six Vision and six No Vision trials for the first dataset, and nine Vision and nine No Vision trials for the second dataset.

### 2.3 Acquisition of Physiological and Behavioral Signals

Both datasets include simultaneously recorded physiological signals (heart rate, skin conductance, respiration, and pupil diameter) and social behavioral cues (body movement, eye contact, and smiling [36]) from both participants.

Physiological signals (sampled at 2048 Hz) of heart rate, electrodermal activity, and respiration rate were recorded using Biosemi electrodes placed at appropriate locations on the body (with signals recorded relative to two other electrodes: common mode sense, the reference electrode, and driven right leg, a feedback circuit that reduces noise by driving the subject's body to a stable potential near the system's electrical zero, both placed on participants’ heads). Specifically, participants’ heart and respiration rates were recorded by attaching a flat touch‐proof Ag‐AgCl active electrode to the thorax (below the right collarbone). Electrodermal activity was measured by placing a flat touch‐proof Ag‐AgCl active electrode on the distal phalange of the left index finger. Pupil size was tracked using a binocular, lightweight eye tracking system (Pupil Labs Core; Pupil Labs, Berlin, Germany [54]). The eye tracking system consisted of three different cameras: Two infrared spectrum eye cameras monitored the two eyes simultaneously (120 Hz sampling frequency; 320 × 280 pixels), while a third (head‐mounted) camera recorded video from the participant's viewpoint (100° fisheye field of view, 30 Hz sampling frequency; 1280 × 720 pixels).

Social behavioral cues were estimated using the video recordings provided by the head‐mounted camera as well as by two additional standalone cameras (SVPRO USB Webcam 5–50 mm Varifocal Lens) mounted on tripod stands. These videos were used to estimate body movement, eye contact, and smiling as detailed in our previous study [36].

### 2.4 Data Analyses

#### 2.4.1 Physiological Measures

##### 2.4.1.1 Heart Rate

The raw electrocardiography (ECG) data were first filtered using a 0.5 Hz high‐pass Butterworth filter (order = 5), followed by powerline filtering (50 Hz). Next, we detected the QRS complexes based on the steepness of the absolute gradient of the ECG signal [55]. From these complexes, the R‐peaks were detected as local maxima (see also [55] for the validation of this algorithm). Heart rate was computed from the period between these peaks and then linearly interpolated (at 2048 Hz). Interpolation was performed using a monotone cubic function that prevents physiologically implausible overshoots or undershoots in the *y*‐direction. All these analyses were performed using functions from the *neurokit2* library [56].

##### 2.4.1.2 Skin Conductance Level

The preprocessing of electrodermal activity involved a low‐pass filter (fourth‐order Butterworth) with a 3 Hz cutoff frequency. The filtered signal was then decomposed into phasic (0.05–3 Hz) and tonic (<0.05 Hz) components. Here, we focused only on the tonic component (sampled at 2048 Hz).

##### 2.4.1.3 Respiration Rate

We estimated the respiratory signal based on heart rate using the “ECG‐derived respiration” measure [57]. For the computation of this measure, the preprocessed heart rate signal described above was band‐pass filtered using a Butterworth filter (0.1−0.4 Hz, order = 2). The sampling rate was kept the same as the original data (2048 Hz). Although this measure is not equivalent to direct measurement, it has been shown to provide a reasonably reliable estimate of respiratory activity, with a low median error (0.015) when validated against photoplethysmography‐based measures [57]. Nonetheless, because this approach represents an indirect proxy of respiratory activity, the resulting respiration indices should be interpreted with due consideration.

##### 2.4.1.4 Pupil Diameter

Pupil diameter (resampled at 30 Hz) was estimated using the open‐source Pupil Player software [54]. The software automatically detects the participant's pupil diameter using two parallel detection methods (2D and 3D pupil detection). Here, we used the 3D pupil detection method as it accounts for perspective. Specifically, this method uses a 3D model of the eye(s) that is updated based on images of the eye. This enables the system to compensate for movements of the Pupil Core eye tracking headset on the participant's face (i.e., slippage).

#### 2.4.2 Social Behavioral Cues

The analysis of behavior followed the same preprocessing we used in a previous study [36]. Here, we briefly describe the critical steps. All behavioral analyses—reconstructing body movement, eye contact, and smiling during the Vision condition—relied on an automated machine learning‐based estimation of face and body landmarks. A deep learning‐based approach was used to extract (i) bidimensional (2D) face landmark locations from videos captured by the head‐mounted cameras of the eye tracker and (ii) 2D full‐body landmarks from videos captured by the two standalone video cameras. Eye contact was approximated as the Euclidean distance between a participant's gaze focus (obtained from the eye tracker) and the center of the partner's face (estimated as the average of the nose landmarks). Overall movement velocity was estimated by taking the first derivative of the 2D positional data (using 25 body landmarks from standalone camera videos) along the *x* and *y* axes separately and then calculating the Euclidean norm of the resulting velocity vectors. Smiling was approximated by computing the aperture of the mouth, which was estimated as the Euclidean distance between the two extreme landmarks on the left and right sides of the lips (extracted from the facial landmarks). Outlying data (defined as data that deviated more than 2.5 standard deviations from their trial‐by‐trial mean) were removed. Further details about these analyses can be found in [36].

#### 2.4.3 Intrapersonal Measures

We evaluated whether the physiological measures (heart rate, skin conductance, respiration rate, and pupil diameter) varied within an individual as a function of visual contact. For this, we computed averages, within each measure, across the trials belonging to the same experimental condition (Vision and No Vision). To determine if these averages differed significantly between conditions, we performed a nonparametric repeated measures multivariate analysis of variance (RM‐MANOVA). This analysis does not rely on assumptions of multivariate normality or specific covariance structures in the data, making it suitable even for data with non‐normal error terms and/or heteroscedastic variances [58, 59]. We estimated the statistical significance of the RM‐MANOVA using a wild bootstrap approach (10,000 iterations) with Rademacher weights (WildBS). If the MANOVA yielded significant results, we conducted paired *t*‐tests to compare the Vision and No Vision for each individual measure and corrected the *p*‐values using a false discovery rate (FDR) correction (across the four measures). FDR correction was chosen as it has been shown to be appropriate and robust for biological data that inherently show dependencies, such as those in the present study [60, 61, 62]. All MANOVA analyses were conducted using the MANOVA.RM package [58] in R.

#### 2.4.4 Interpersonal Measures

We estimated the extent to which the physiological measures synchronized across the two individuals forming a dyad. Synchrony was operationalized using Pearson's correlations of the data time series spanning the full trial duration (i.e., 2 min), as in prior studies [63, 64, 65, 66]. These correlation coefficients were then averaged across trials, resulting in one coefficient per dyad and condition. To assess differences in synchrony between conditions, we performed a nonparametric RM‐MANOVA. Significant results from MANOVA were followed up with paired *t*‐tests for each physiological measure.

Next, we assessed whether synchrony emerged from dyad‐specific interactions rather than from spurious correlations (e.g., those arising from shared signal properties or autocorrelations that reflect general sample trends rather than true dyadic interaction) [29, 67, 68]. To do so, we created surrogate dyads by randomly pairing individuals who had not interacted with each other (dyad‐composition randomization analysis as in [8, 36, 68, 69, 70, 71, 72]). Specifically, for each participant and condition within a dyad, we generated all possible pairings between that participant's data and data from other participants in the same condition drawn from different dyads. This resulted in 22 combinations per condition per participant in the first dataset and 14 combinations in the second dataset. Importantly, the randomization was performed in a condition and order‐specific manner: for example, the first trial belonging to the vision condition of one participant was paired only with the first trial belonging to the vision condition of another participant. This ensured that (i) Vision and No Vision conditions were compared separately, and (ii) the temporal order of trials within conditions was maintained. By preserving condition structure, this procedure provides a conservative and robust estimate of chance‐level synchrony. The correlations were then averaged for each participant and condition, yielding a single pseudo‐correlation value per dyad (following procedures similar to [41, 44, 73]). Thus, each dyad was assigned one representative pseudo‐correlation value reflecting chance‐level coupling for that dyad. Finally, these pseudo‐correlations emerging from the surrogate dyads were statistically compared (using paired tests) with the correlations derived from the actual interacting partners (i.e., real dyads) [74]. We adopted this approach particularly because the data were drawn from different datasets with partially differing condition structures and trial numbers. Additionally, to evaluate the robustness of our findings to statistical approaches that explicitly account for dependency in the data, we replicated the analyses using linear mixed‑effects models applied to individual dyadic synchrony estimates. In these models, pair type (real vs. surrogate), condition (Vision vs. No Vision), and their interaction were included as fixed effects, while crossed random intercepts were specified for the two members of each dyad to account for repeated participation across dyads  [75, 76].

Furthermore, we examined interpersonal synchrony in behavioral measures, focusing selectively on the Vision condition, as the behavioral cues were not recorded during the No Vision condition (i.e., the occluder prevented the cameras from recording the partner). Consistent with the previous analyses on physiological data, behavioral synchrony was parameterized as Pearson's correlations between the two participants’ time series across the entire trial. For each behavioral measure, the correlation coefficients were then averaged across trials, resulting in one coefficient per dyad. To evaluate whether these coefficients were significantly higher than chance levels, we conducted two analyses. First, we compared the coefficients using one‐sample *t*‐tests versus a test value of 0 (signifying a chance‐level correlation). Second, we generated surrogate dyads as above by pairing participants who did not interact with each other. We corrected the resulting *p*‐values obtained using an FDR correction [77].

#### 2.4.5 Integration of Physiological and Behavioral Data

We used a Bayesian hierarchical linear regression to test the association between synchrony of physiological measures and of social behavioral cues (following the approach described in [36]). This modeling approach was used because it allows for meaningful parameter estimation at multiple levels of the data structure while also integrating information both within and across these levels [78]. The Bayesian framework was chosen specifically for its ability to represent parameter uncertainty directly through posterior distributions, offering interpretable estimates without relying on sampling distributions from null hypotheses, as required in frequentist approaches [79, 80, 81]. We tested the association between physiological and behavioral synchrony selectively during the Vision condition since the behavioral cues were recorded only during the Vision condition (as described above).

Since participants produced behaviors spontaneously, we conducted the analysis at a sample‐by‐sample resolution to preserve the temporal dynamics that would have been lost through averaging. Accordingly, we estimated sample‐by‐sample IPS and behavioral synchrony by computing indexes of instantaneous correlation between the two partners’ time series [82], which were down‐sampled to 10 Hz. Instantaneous correlations were computed by first subtracting the mean of a time series and normalizing it to a norm vector. Each time point of this normalized data was then represented as a point in a 2D space with data from the two participants on the two axes. The correlation index was computed as the smaller angle subtended by this vector to a line orthogonal to the equality line. The instantaneous correlation was finally max‐normalized (range 0–1).

Next, we performed a hierarchical regression using the obtained indices of instantaneous correlation. This entails a partial‐pooling (or multilevel approach) that estimates the group coefficients while allowing the subject‐level coefficients to vary [83]. We modeled the average of group‐level coefficients as random variables with normal distributions centered at 0, standard deviations of 100, and the group standard deviations as half‐normal distributions with standard deviations of 5 (see [36] for more details). The overall error was modeled as a half‐Cauchy log‐likelihood with a scale parameter of 5. The subject‐level coefficients were modeled as normal distributions with the mean centered at the group‐level average and the standard deviation at the group‐level standard deviation. The posterior distribution was sampled using the Markov chain Monte Carlo sampling algorithm “No‐U‐Turn Sampler” [84]. We sampled 1000 samples on four separate chains and used a tuning parameter of 1000 samples. We performed the model selection using the Pareto‐smoothed importance sampling leave‐one‐out (PSIS‐LOO) cross‐validation method [85]. Finally, we determined the significance of the group‐level coefficients based on the overlap of their estimated posterior distributions with the test value of 0. A difference of less than 5% in the posterior distribution overlap (Pp|D) was considered significant.

## 3 Results

### 3.1 Intrapersonal Measures: Physiological Changes Driven by Visual Contact

**FIGURE 2:**
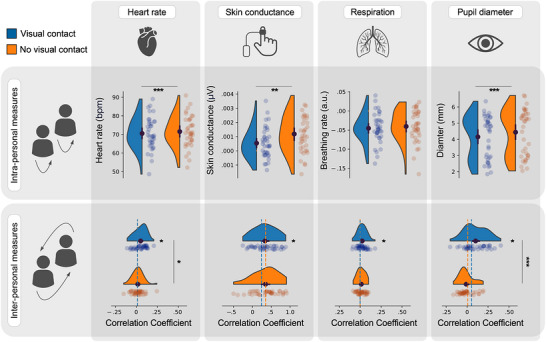
Effect of visual contact on intra and interpersonal measures. Top: Intrapersonal measures. Violin plots display averages (each dot represents the average of two participants forming a dyad), separately for Vision (blue) and No Vision (orange) conditions. Physiological measures such as heart rate, skin conductance, and pupil diameter (but not respiration rate) were significantly lower when the participants could see each other compared to when they could not. Bottom: Interpersonal measures. Raincloud plots display indices of interpersonal synchrony (Pearson's correlation coefficients, each dot represents a dyad). The dashed vertical lines index chance correlation coefficients obtained following a randomization of dyad composition analysis, separately for Vision (blue dashed line) and No Vision (orange dashed line) conditions. Synchrony across all measures exceeded chance levels only in the Vision condition. Moreover, heart rate and pupil diameter synchrony were significantly higher in the Vision than in the No Vision condition. ***p<0.001; **p<0.01; *p<0.05.

We evaluated whether body physiology changed within individuals as a function of visual contact. A MANOVA on averaged intrapersonal measures yielded a significant effect of Vision (Wald‐type statistic W = 61.7, *df* = 4, *p* < 0.001). This indicated that, overall, physiological measures were lower when participants could see each other compared to when they could not (Figure 2, top). Follow‐up paired *t*‐tests revealed that the above‐described effect was driven by heart rate, skin conductance, and pupil diameter—but not respiration rate (Table 1). Together, these results suggest that visual contact with the partner leads to lower levels of arousal, as indexed by slower heart rate, lower skin conductance, and smaller pupil diameter.

**TABLE 1:** Summary statistics for intra and interpersonal measures.

	Statistic (*t*‐value)	*p‐*value (FDR corrected)	Effect size (Cohen's *d*)
**Intrapersonal measures (Vision condition vs. No Vision condition)**			
Heart rate	3.9	**<0.001**	0.6
Skin conductance	3.6	**0.001**	0.6
Respiration	0.9	0.4	0.2
Pupil diameter	5.6	**<0.001**	0.9
**Interpersonal measures (Vision condition vs. No Vision condition)**			
Heart rate	2.3	**0.03**	0.4
Skin conductance	−0.3	0.8	−0.05
Respiration	0.9	0.4	0.2
Pupil diameter	4.5	**<0.001**	0.7
**Interpersonal measures (real dyads vs. surrogate dyads—Vision condition)**			
Heart rate	3.1	**0.03**	0.5
Skin conductance	2.4	**0.04**	0.4
Respiration	2.6	**0.04**	0.4
Pupil diameter	2.8	**0.03**	0.5
**Interpersonal measures (real dyads vs. surrogate dyads—No Vision condition)**			
Heart rate	0.29	0.9	0.05
Skin conductance	0.06	1.0	0.01
Respiration	1.8	0.1	0.3
Pupil diameter	−1.3	0.3	−0.2

*Note*: Significant effects are shown in bold.

### 3.2 Interpersonal Measures: Synchrony Changes Driven by Visual Contact

We first evaluated whether IPS changed as a function of visual contact. Even though IPS coefficients were overall small in magnitude (Table 2), they were reliably modulated by vision, yielding medium‐sized effects (Table 1). A MANOVA on these data revealed a significant effect for Vision (W = 27.54, *df* = 4, *p* < 0.001), and follow‐up paired *t*‐tests revealed that this effect was driven by differences in heart rate and pupil diameter (Table 1). Hence, when participants could see each other, their heart rate and pupil diameters were more synchronized compared to when they could not see each other (Figure 2, bottom).

**TABLE 2:** Interpersonal measures (coefficients): Condition‐wise means and standard deviations.

	Average	Standard deviation
**Interpersonal measures (Vision condition)**		
Heart rate	0.05	0.07
Skin conductance	0.3	0.3
Respiration	0.02	0.06
Pupil diameter	0.09	0.1
**Interpersonal measures (No Vision condition)**		
Heart rate	0.02	0.07
Skin conductance	0.4	0.4
Respiration	0.01	0.05
Pupil diameter	−0.01	0.07

To understand whether IPS emerged from dyad‐specific interactions, as opposed to spurious correlations emerging from similarities in data properties [29, 67], we performed a randomization of dyad composition analysis. For this, we generated surrogate dyads of participants by pairing individuals who did not interact with each other (as in [8, 36, 68, 69, 70, 71]). We then compared IPS across real dyads and such surrogate dyads. This analysis revealed that all measures of physiological synchrony were significantly higher in real dyads compared to surrogate dyads, notably only during Vision conditions (FDR corrected across eight contrasts comparing real vs. surrogate dyads across all Vision and No Vision conditions *p*s < 0.05) (Table 1 and Figure 2, bottom). Furthermore, complementary linear mixed‑effects analyses that explicitly accounted for dependency in the surrogate data yielded fully consistent results, confirming the robustness of these effects (Tables S1 and S2).

Together, these results indicate that physiological synchrony is generally enhanced by mere visual contact. Yet, some measures appeared to be more robustly synchronized than others. In particular, synchrony of heart rate and pupil diameter was enhanced both with respect to chance‐level synchrony in surrogate dyads and with respect to the No Vision condition, while synchrony of skin conductance and respiration was only enhanced with respect to chance‐level synchrony.

### 3.3 Relationship Between Physiological Synchrony and Behavioral Synchrony

The results above indicate that physiological synchrony reflects dyad‐specific modulations. To explore the behavioral correlates of these modulations, we examined whether physiological synchrony co‐occurred with the emergence of synchronized social behaviors [36, 37].

**FIGURE 3:**
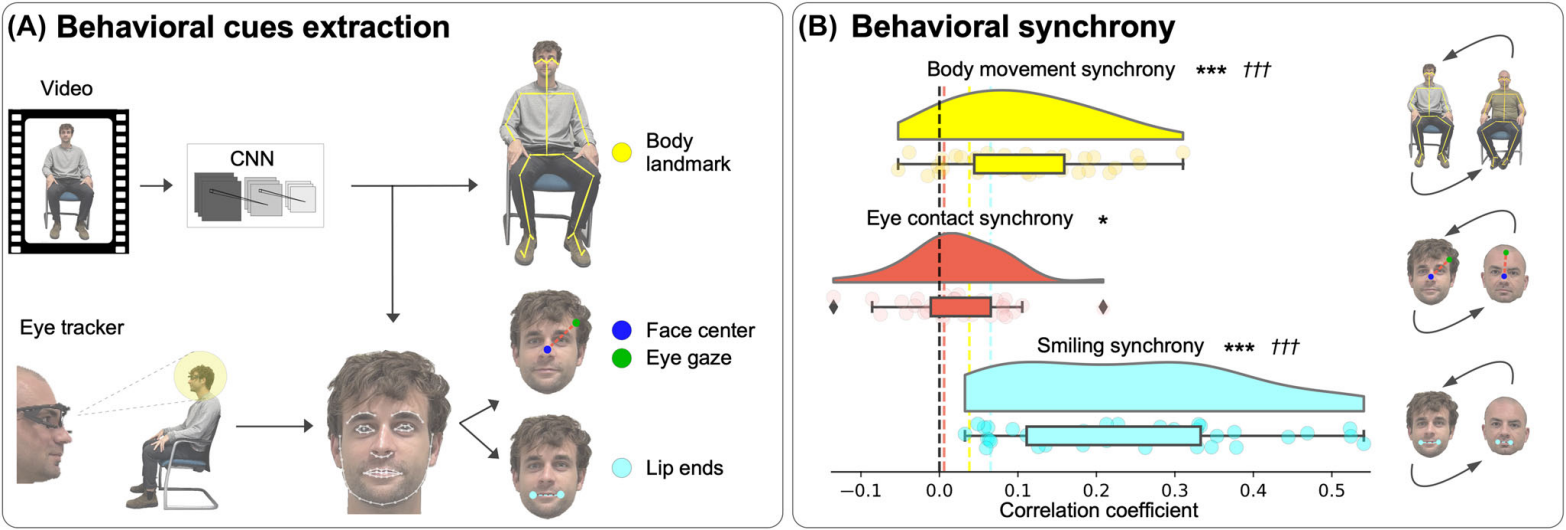
Interpersonal synchronization of behavioral measures. (A) Behavioral cues extraction. Three behaviors were automatically extracted using a trained multistage convolutional neural network (CNN). This network was first used to estimate 25 body and 70 face landmarks. The body landmarks yielded estimates of whole‐body movement, while the face landmarks yielded estimates of smiling behavior or were combined with eye tracking data to yield estimates of eye contact. (B) Behavioral synchrony. Pearson's correlation coefficients were used to estimate spontaneous synchronization of behavioral cues across members of a dyad. The analysis revealed that all behavioral cues spontaneously synchronized (comparing the coefficients to a test value of 0; FDR corrected *p*s <0.05). However, synchronization appeared to be more robust for spontaneous body movements and smiling behavior compared to eye contact (as indicated by a *t*‐test vs. surrogate chance level *p*‐value > 0.05). Hence, participants robustly synchronized their spontaneous body movements and smiling behavior. ***p<0.001; *p<0.05 versus test value 0; ^†††^ *p* < 0.001 versus surrogate chance level. The black dashed line indicates a coefficient of 0, whereas the colored dashed lines indicate the surrogate‐derived chance level. Diamond markers represent outliers.

To investigate this, we first extracted measures of spontaneous body movement, eye contact, and smiling during the Vision condition (see Section 2). In line with our previous work [36, 37], we confirmed that, in addition to physiological measures, behavioral measures also showed interpersonal synchrony during visual contact (synchrony in body movement—*t*‐test vs. 0, *t*(36) = 7.0, FDR corrected *p* <0.001, *t*‐test vs. surrogate dyads *p* <0.001; eye contact—*t*(36) = 2.2, FDR corrected *p* = 0.034, *t*‐test vs. surrogate dyads *p* = 0.15; smiling—*t*(36) = 9.8, FDR corrected *p* <0.001, *t*‐test vs. surrogate dyads *p* <0.001; Figure 3). That is, participants spontaneously synchronized their body movements and smiling behavior, while evidence for eye contact was less robust. Next, we computed instantaneous synchrony indices [36, 82] for both physiological and behavioral measures to capture how synchrony evolved over time. We then combined these time series to examine their relationship, estimating the association between IPS and synchronized behaviors using a Bayesian hierarchical (partial‐pooling) linear regression model, where instantaneous IPS was predicted from instantaneous behavioral synchrony.

**FIGURE 4:**
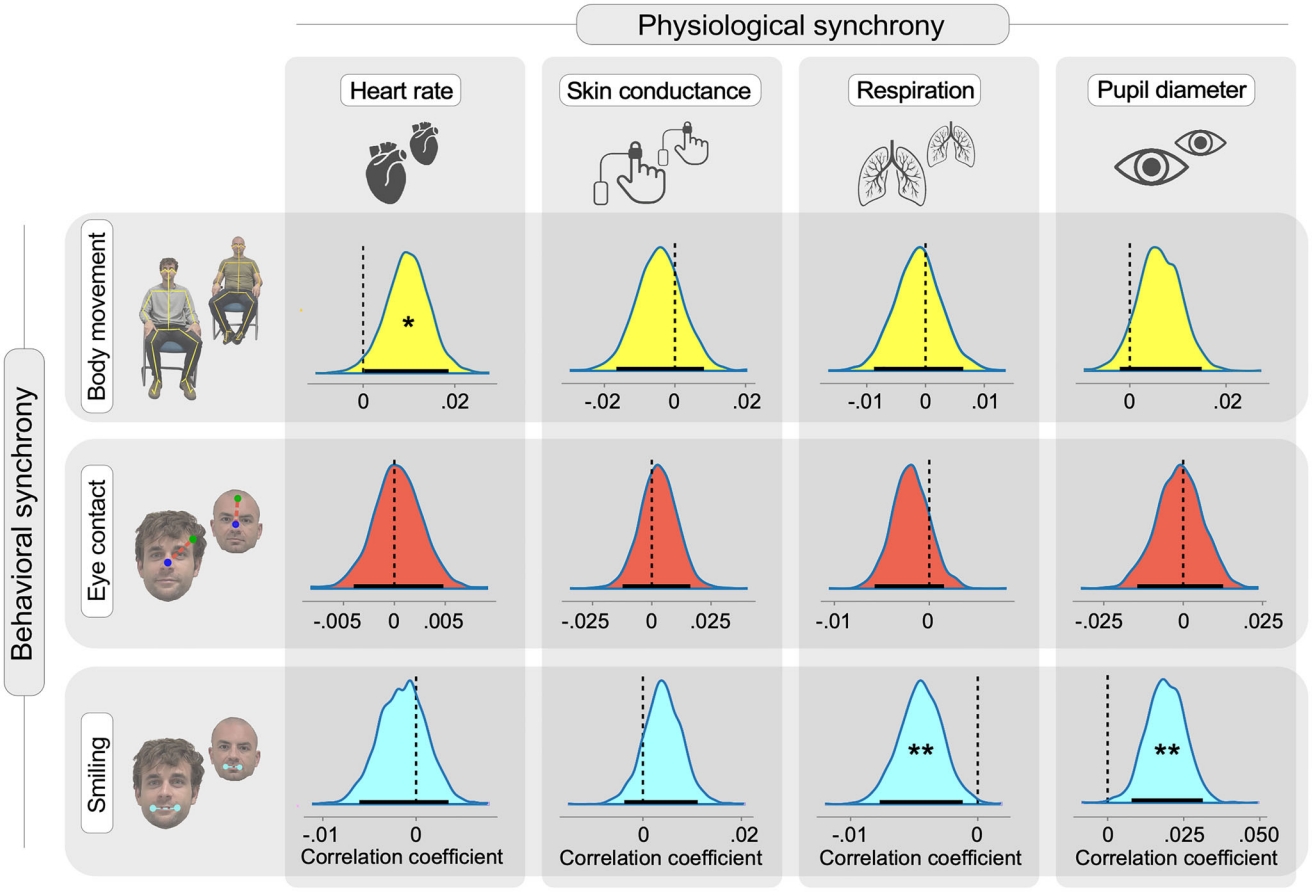
Relationship between physiological and behavioral synchrony. Posterior distributions of correlation coefficients are shown for each behavioral–physiological synchrony pair. Heart rate synchrony was positively associated with body movement synchrony, and pupil diameter synchrony with smiling synchrony. Respiration synchrony was negatively related to smiling synchrony. The black vertical dashed line indicates zero; dark horizontal bars denote the 94% highest density interval (HDI). Error bars represent standard errors. *p < 0.05; **p < 0.01.

We estimated models including all behavioral synchronies as predictors of IPS, with separate models analyzed for each physiological measure. Model comparisons using PSIS‐LOO cross‐validation showed that synchronized social behavior models explained the variance in physiological synchrony better than their respective null models (Table 3). Examining the individual regression models, we found that heart rate synchrony significantly predicted body movement synchrony, while pupil diameter synchrony predicted smiling synchrony (Figure 4 and Table 4). Additionally, we observed a negative relationship between respiration synchrony and smiling synchrony.

**TABLE 3:** Model performance for the relationship between physiological synchrony and behavioral synchrony.

Physiological measure	Model	EPLD	EPLD standard error	Weight
Heart rate synchrony	Null	−35,402.1	193.5	0
	Behavioral synchronies	−34,997.1	194.5	1
Skin conductance synchrony	Null	−36,772.8	234.3	0
	Behavioral synchronies	−36,085.8	236.2	1
Respiration rate synchrony	Null	−33,326.6	196	0
	Behavioral synchronies	−33,117.5	196.6	1
Pupil diameter synchrony	Null	−242,978	274.2	0.2
	Behavioral synchronies	−242,734	274.6	0.8

Abbreviation: EPLD, approximated expected log pointwise predictive density. Weight: These weights can be roughly interpreted as the probability, given the data, that a specific model is the true model (among the models being compared).

**TABLE 4:** Correlation coefficients for the relationship between physiological and behavioral synchrony.

Physiological measure	Parameter	Mean estimate	P_p|D_ (parameter <0)	94% HDI	*R*‐hat value
Heart rate synchrony	Body movement synchrony	0.01	**0.03**	[0 0.019]	1
	Eye contact synchrony	0	0.5	[−0.004 0.005]	1
	Smiling synchrony	−0.001	0.7	[−0.006 0.003]	1
Skin conductance synchrony	Body movement synchrony	−0.004	0.8	[−0.017 0.008]	1
	Eye contact synchrony	0.003	0.4	[−0.012 0.017]	1
	Smiling synchrony	0.004	0.2	[−0.004 0.011]	1
Respiration synchrony	Body movement synchrony	−0.001	0.6	[−0.009 0.006]	1
	Eye contact synchrony	−0.002	0.8	[−0.006 0.002]	1
	Smiling synchrony	−0.004	**1**	[−0.008 −0.001]	1
Pupil size synchrony	Body movement synchrony	0.006	0.09	[−0.002 0.015]	1
	Eye contact synchrony	−0.001	0.6	[−0.015 0.013]	1
	Smiling synchrony	0.019	**0.002**	[0.008 0.031]	1

*Note*: An *R*‐hat value that deviates from the value of 1 reflects issues with the model convergence. Significant effects are shown in bold. Heart rate synchrony was linked to body movement synchrony, pupil size synchrony to smiling synchrony, and respiration synchrony showed a negative relationship with smiling synchrony.

Abbreviations: Mean estimate, average coefficient estimates from the posterior distribution; Pp|D, posterior distribution overlap of the parameter higher than the test value of 0; *R*‐hat value, *R*‐hat convergence diagnostic compares the between‐ and within‐chain estimates for model parameters; 94% HDI, 94% highest density interval.

## 4 Discussion

We demonstrated that visual contact between two individuals is sufficient to synchronize the activity of their autonomic nervous systems. Specifically, we found that visual contact is sufficient to robustly synchronize heart rates, pupil diameters, and, to a lesser degree, breathing rates and skin conductance. We also demonstrated that the interpersonal synchrony of these physiological measures co‐occurred with the interpersonal synchrony of social behaviors. In particular, synchrony of pupil diameters co‐occurred with synchronized smiling, while synchrony of heart rates co‐occurred with synchrony of spontaneous body movements. Together, these results imply that real‐time visual contact between two individuals leads to the temporal alignment of both autonomic and behavioral processes, and that these processes are interrelated.

### 4.1 Interpersonal Physiological Synchrony

Previous research has already established that social interaction leads to the interpersonal alignment of the physiological measures tested here—namely, heart rate [4, 44, 51, 86, 87, 88], pupil diameter [16, 17, 89], breathing rate [11, 90, 91], and skin conductance [8, 92, 93, 94]. Our first contribution is that this phenomenon can be observed even in the context of a minimalistic social interaction, such as mere visual contact with another person. This observation is noteworthy because it sheds light on the origins of IPS. While previous research has attributed the emergence of IPS to the specific nature of the social tasks performed by participants [41, 87, 94], here we show that such tasks—often conflated with the visual exchange of social cues—might not be necessary for the synchrony to emerge.

But how does IPS emerge from mere visual contact? One possibility is that individuals directly perceived or inferred their partner's physiological signals and, either consciously or unconsciously, aligned their own accordingly. Some previous studies support this idea, showing that certain physiological signals can indeed be perceived directly. For instance, Galvez‐Pol et al. [95] demonstrated that humans can partially detect others’ cardiac rhythms simply by observing their faces. Similarly, Harrison et al. [96] found that seeing faces with smaller pupil sizes leads to corresponding pupil constriction in the observer (i.e., pupil mimicry). Therefore, it is possible that physiological signals, such as heart rate and pupil diameter—both robustly synchronized in our study—may be accessible to partners through visual cues such as changes in facial redness or subtle pulsatile movements in the face, head, or eyes. At the same time, it is possible that perceiving just one physiological signal (e.g., heart rate) in a partner could be sufficient to indirectly synchronize other signals (e.g., breathing rate, pupil size, and skin conductance), especially given that all of these measures are manifestations of a shared underlying autonomic regulatory system [97]. More generally, accounts of emotional and physiological contagion emphasize how visual information during social interaction can support alignment of internal states [98], which may be relevant for interpreting the present findings.

Alternatively, physiological synchrony might have emerged through the exchange of behavioral cues. In this view, visually perceiving a partner's behavior—such as facial expressions and body movements—would provide information about their internal physiological state. These cues may, in turn, elicit corresponding changes in the observer's behavior and autonomic responses, ultimately giving rise to IPS. In support of this account, extensive prior research has demonstrated that observing others’ behavior often leads to behavioral synchrony [99, 100, 101, 102, 103], even when those behaviors are spontaneous [37]. Notably, such behavioral alignment is frequently accompanied by physiological synchrony [23, 24, 48, 104]. We empirically tested this possibility in our study, which we directly discuss further below.

### 4.2 Multiple Physiological Measures and Timescales

Another novel contribution of our work is that, unlike most of the previous studies on this topic, which measured one physiological measure at a time, here we measured four physiological measures simultaneously. This is not only a technical advancement, but also something that permitted us to make a novel observation: some measures appeared to be more robustly synchronized than others. In particular, the IPS of heart rate and pupil diameter was enhanced both with respect to chance‐level synchrony (i.e., surrogate dyads) and with respect to the No Vision condition, while the IPS of skin conductance and respiration was only enhanced with respect to chance‐level synchrony. What could explain this finding?

A plausible explanation might be found in the markedly different timescale of these measures [105]. Since skin conductance (which changes over 10–60 s) and respiration (with 3–6 s cycles) are much slower than pupil responses (0.5–4 s) and heart rate (which varies beat‐to‐beat, around every second), their interpersonal synchronization may produce longer‐lasting effects that extend beyond the current trial durations. In other words, even though the vision of a partner favored synchrony of all physiological measures, it is possible that, once established, synchrony carried on throughout the following trials associated with no vision of a partner. This hypothesis could be tested by repeating our experiment with longer trials or longer inter‐trial intervals.

### 4.3 Relationship Between Intrapersonal and Interpersonal Physiological Measures

Besides assessing how physiological measures synchronized interpersonally, we also assessed how they changed intrapersonally, that is, at the level of the individuals, irrespective of their partners. This was meant to shed light upon the arousing state of the participants, and to indirectly inform us on the mechanisms supporting the establishment of synchronized autonomic activities.

This analysis revealed that visual contact with the partner led to a slower heart rate, lower skin conductance, and smaller pupil diameter. Breathing rate was not modulated by the vision manipulation. These results indicate that participants’ arousal generally decreased when they could see each other. This could possibly be explained in terms of the vision condition being relatively more comfortable, and/or the no vision condition being more stressful [106, 107]. Considering that participants forming a dyad were familiar with each other (see Section 2), this hypothesis might be further supported by evidence showing that seeing familiar faces can indeed reduce stress and negative affect [108, 109]. Unfortunately, we cannot test this hypothesis because we did not collect direct measures of stress or anxiety from the participants. As such, these explanations remain largely speculative.

Furthermore, the intrapersonal changes might inform on the mechanisms through which interpersonal synchrony was reached. Rhythmic signals, such as the heart rate, slowed down during vision. In some cases, this can favor higher correlations between the measures due to low‐frequency signals tending to be smoother and less noisy, and better reflecting sustained co‐fluctuations over time. In the current study, we observed that heart rate indeed slowed down during the vision condition. However, the synchrony analysis of equally slow signals from real and surrogate dyads showed higher synchrony in real dyads, suggesting that the observed synchrony cannot be explained solely by a general slowing of heart rate.

Another intrapersonal factor that may influence interpersonal synchrony is the autocorrelation inherent in the physiological signals [110]. Indeed, physiological signals, especially heart rate, often exhibit autocorrelation. However, it is unlikely that autocorrelation accounted for the vision effect in the current study. If autocorrelation were the primary driver of coupling, then we should expect to replicate our findings also for surrogate, randomized dyads, but this was not the case. Specifically, when IPS was computed between non‐interacting partners while keeping condition and trial order constant (see Section 2), we did not observe a significant effect of vision on IPS. This suggests that the interpersonal synchrony observed in the current study likely reflects real‐time coupling rather than shared signal properties or intrinsic autocorrelation.

Overall, we highlight the importance of considering how intrapersonal changes of physiological measures might facilitate or modulate the emergence of synchronized activities.

### 4.4 Relationship Between Interpersonal Physiological and Behavioral Measures

Previous research on IPS has mostly focused on investigating how IPS changes as a function of experimental conditions [1, 111]. Very little attention has been paid to other co‐changing variables, such as behavioral ones (cf. [24, 34]). This issue, however, is central to the understanding of the nature of IPS because behavior leads to salient changes in the environment that are perceivable by a partner. Of course, it could be argued that some of the physiological measures we collected might also be perceived by partners, certainly breathing and pupil size, and there is also work suggesting that heart rate can be inferred through vision of a person's face [95]. Yet, social behaviors such as body movement and smile are certainly salient means through which two individuals transfer information between one another, possibly leading to synchronized internal states.

In line with our previous work demonstrating that interneural synchrony co‐occurs with synchrony of social behavior [36], here we tested if the latter also accompanies physiological synchrony. We found some support for this hypothesis. Specifically, pupil diameter synchrony was associated with smiling synchrony, while heart rate synchrony was associated with synchronized body movements. The association between smiling and pupil diameter could be generally explained by the fact that they can both signal (and trigger) positive affect [112]. For instance, it could be argued that observing smiles led to increases in pupil diameter. Alternatively, and more parsimoniously, smiling and pupil diameter might be correlated intrapersonally. Specifically, an individual's smile could trigger concurrent changes in their pupil diameter, which might then lead to correlations at the interpersonal level.

The association between heart rate synchrony and body movement synchrony may be physiologically explained by the relationship between movement and heart dynamics, which is present even during small movements [113, 114]. The association between these two types of synchronies is consistent with prior studies that observed similar effects—although in contexts involving instructed actions. For instance, Noy et al. [63] reported an association between heart rate synchrony and periods of coordinated hand movements during an imitation task. Our results extend these findings by demonstrating that such physiological–behavioral associations can emerge spontaneously, even in the absence of explicit instructions to move.

Additionally, we also observed a negative relationship between respiration rate synchrony and smiling synchrony. While it is difficult to interpret this result, it is possible that this relationship reflects the impact of facial movements on breathing [115, 116]. Indeed, as smiling involves changes in the orofacial musculature and increases the oral cavity aperture, it may, in turn, influence the volume of air inhaled during respiration. Previous research has indeed shown how facial actions similar to smiling can increase the volume of inhaled air and disrupt the normal rhythm of breathing [115]. Here, differences in smile intensity between individuals (e.g., broad vs. subtle smiles) may have unevenly influenced their breathing patterns, causing a mismatch in rhythms and reducing respiratory synchrony. This remains a speculative explanation, which, however, could be tested in the future along with other potential accounts.

More generally, our approach and findings add to the growing literature investigating the interdependence between physiological, behavioral, and neural signals. Previously, we reported a relationship between behavioral and neural synchrony [36], while here we report one between behavioral and physiological synchrony. What is missing is a holistic approach, capturing all measures simultaneously and investigating not only if they occur together, but whether some of them mediate others. We anticipate a dynamic interdependence between these measures [117] and believe this should be explored in the future using computational models. At the same time, to effectively test the directionality of these effects and fully clarify the causal relationship between social behavior, neural, and physiological synchrony, these models would need to be complemented by causal manipulations, that is, experimentally manipulating synchrony in one domain (e.g., physiology) and then observing its effect on another domain (e.g., behavior or neural activity) [118, 119, 120, 121, 122]. Such an integrated, multimodal approach will help clarify the mechanisms by which humans become attuned to one another.

## Author Contributions

Atesh Koul: Conceptualization, formal analysis, investigation, methodology, software, visualization, data curation, writing – original draft, writing – review and editing. Giacomo Novembre: Conceptualization, investigation, methodology, visualization, writing – original draft, writing – review and editing, supervision, project administration, funding acquisition.

## Conflicts of Interest

The authors declare no conflicts of interest.

## Supporting information




**Supplementary Tables**: nyas70322‐sup‐0001‐TableS1‐S2.docx

## Data Availability

Raw data that support the findings of this study are available in the IIT dataverse repository: https://doi.org/10.48557/1IUJVN. Data analysis libraries and scripts are available on GitHub: https://github.com/ateshkoul/physio_synch.
