# Supplementary material for: Interpersonal Synchronization of Autonomic Physiology via Mere Visual Contact
Source: Ann N Y Acad Sci. 2026 Jun 16;1560(1):e70322. doi: 10.1111/nyas.70322 (PMC13271046; doi:10.1111/nyas.70322)
Supplement: Supplementary file 1 — Supplementary Tables: nyas70322‐sup‐0001‐TableS1‐S2.docx [file NYAS-1560-0-s001.docx]

Supplementary information for

**Interpersonal synchronization of autonomic physiology via mere visual contact**

Atesh Koul^1*^ & Giacomo Novembre^1*^

^1^ Neuroscience of Perception and Action Lab, Italian Institute of Technology, Rome, Italy.

*Correspondence to: Atesh Koul and Giacomo Novembre

Italian Institute of Technology (IIT),

Viale Regina Elena 291, 00161 Rome, Italy

Email: [atesh.koul@iit.it](mailto:atesh.koul@iit.it), [giacomo.novembre@iit.it](mailto:giacomo.novembre@iit.it)

Phone: +39 06 49255199

**Supplementary Table S1. Linear mixed‑effects ANOVA results for surrogate analyses.** To evaluate the robustness of our findings to statistical approaches that explicitly account for dependency in the data, we replicated the surrogate‑dyad analyses using linear mixed‑effects models applied to dyadic synchrony estimates. Models included fixed effects for pair type (real vs. surrogate), condition (Vision vs. No Vision), and their interaction, with crossed random intercepts for the two members of each dyad. Significant effects are shown in bold.

| Physiological measure | Effect | F value (Type III) | P value |
| --- | --- | --- | --- |
| Heart rate | Pair type | 7.9 | **0.005** |
|  | Condition | 5.3 | **0.022** |
|  | Pair type × Condition | 5.5 | **0.019** |
| Skin conductance | Pair type | 4.9 | **0.028** |
|  | Condition | 7.3 | **0.007** |
|  | Pair type × Condition | 2.9 | 0.090 |
| Respiration | Pair type | 12.1 | **<0.001** |
|  | Condition | 1.0 | 0.31 |
|  | Pair type × Condition | 1.0 | 0.31 |
| Pupil diameter | Pair type | 1.5 | 0.28 |
|  | Condition | 54.5 | **< 0.001** |
|  | Pair type × Condition | 6.5 | **0.011** |

**Supplementary Table S2. Post‑hoc contrasts from linear mixed‑effects models.** Post‑hoc contrasts (estimated marginal means) comparing real and surrogate dyads within each condition, derived from the same linear mixed‑effects models used above (Supplementary Table S1). Models were applied to individual dyadic synchrony estimates and included crossed random intercepts for the two members of each dyad. Significant effects are shown in bold.

| Physiological measure | Estimate | P value |
| --- | --- | --- |
| **Inter-personal measures (Real dyads vs surrogate dyads – Vision condition)** | | |
| Heart rate | 3.6 | **<0.001** |
| Skin conductance | 2.8 | **0.006** |
| Respiration | 3.2 | **0.002** |
| Pupil diameter | 2.7 | **0.008** |
| **Inter-personal measures (Real dyads vs surrogate dyads – No Vision condition)** | | |
| Heart rate | 0.3 | 0.75 |
| Skin conductance | 0.4 | 0.72 |
| Respiration | 1.7 | 0.08 |
| Pupil diameter | −0.9 | 0.35 |
